# Short-chain PFAS exposure during gestation and breastfeeding alters learning and memory in adulthood: possible mechanisms related to brain development

**DOI:** 10.3389/ftox.2025.1702330

**Published:** 2026-01-08

**Authors:** Luca Lorenzini, Marzia Moretti, Claudia Zanardello, Federica Gallocchio, Vito A. Baldassarro, Alessandra Moressa, Lorenzo Zanella, Michele Sannia, Greta Foiani, Corinne Quadalti, Maura Cescatti, Valentina Burato, Margherita Soncin, Marzia Mancin, Luciana Giardino, Franco Mutinelli, Marta Vascellari, Laura Calzà

**Affiliations:** 1 Department of Veterinary Medical Sciences, University of Bologna, Bologna, Italy; 2 Interdepartmental Centre for Industrial Research in Health Sciences and Technologies - CIRI Health Sciences and Technologies, University of Bologna, Bologna, Italy; 3 Istituto Zooprofilattico Sperimentale delle Venezie, Padova, Italy; 4 IRET ETS Foundation, Bologna, Italy; 5 Department of Pharmacy and Biotechnology, University of Bologna, Bologna, Italy

**Keywords:** cognition, GenX, hippocampus, neurodevelopment, neuroinflammation, perfluorobutanoic acid, risk assessment, short-chain perfluoroalkyl substances

## Abstract

**Introduction:**

Exposure to long-chain perfluoroalkyl substances (PFASs) during development has been consistently associated with cognitive impairment and behavioural changes in humans. These concerns have led to regulatory restrictions and a shift towards short-chain PFASs as alternatives. However, experimental evidence on the neurodevelopmental impact of short-chain PFASs remains scarce, despite their increasing detection in drinking water and human biomonitoring studies.

**Methods:**

This study provides the first experimental evidence of the neurodevelopmental toxicity of maternal exposure to the short-chain PFASs GenX and PFBA, administered before mating, throughout gestation, and during lactation.

**Results:**

In a rat model, offspring from exposed dams displayed significant impairments in spatial learning and cognitive flexibility in the Morris water maze. Mechanistic investigations on PFBA exposure *ex vivo* revealed delayed neuronal maturation, reduced expression of MAP2, PSD95 and VGLUT. Impaired neurogenesis persisted into adulthood in the hippocampus, as shown by upregulation of nestin and downregulation of doublecortin, together with dysregulated expression of neuroinflammatory genes in the hippocampus for both tested molecules.

**Discussion:**

Our findings indicate that even short-chain PFASs, currently considered safer substitutes, may disrupt brain development, leading to persistent neuroinflammation and impaired cognitive function. These results highlight an urgent need to reassess the developmental safety of short-chain PFASs and to include neurodevelopmental endpoints in future risk assessments and regulatory policies.

## 1 Introduction

Environmental pollution is increasingly recognized as a health risk not only from acute, high-dose exposures but also through low-level accumulation of persistent organic pollutants (POPs), the so-called “forever chemicals.” Among them, per- and polyfluoroalkyl substances (PFAS) form a large group (>9,000) of highly stable fluorinated compounds widely used since the 1940s for their non-stick, waterproof, and stain-repellent properties in food packaging, textiles, and consumer products (EFSA Panel on Contaminants in the Food Chain, 2020; Giesy and Kannan, 2001). Their persistence has resulted in widespread contamination and bioaccumulation in wildlife and humans (Gallocchio et al., 2022; De Silva et al., 2021).

Long-chain PFAS (“legacy PFAS”) include sulfonates with ≥6 carbons and carboxylic acids with ≥8 carbons. Exposure to these compounds has been associated with multiple toxic effects, including developmental, reproductive, hepatic, immune, endocrine, and neurotoxic outcomes (Fenton et al., 2021; Steenland and Winquist, 2021). Importantly, several epidemiological studies have reported associations between prenatal PFAS exposure and altered child neurodevelopment. The Canadian MIREC cohort linked higher prenatal PFAS levels to reduced performance IQ in boys (Goodman et al., 2023), while a Chinese study reported adverse neurodevelopmental status at 2 years of age (Luo et al., 2022). Although some studies found mixed results (Lenters et al., 2019), the overall evidence suggests that fetal and early-life exposure may affect cognition and behavior in children, supporting the need for mechanistic experimental data. Based on this evidence, perfluorooctane sulfonic acid (PFOS), perfluorooctanoic acid (PFOA), and perfluorohexane sulfonic acid (PFHxS) have been listed under the Stockholm Convention (2009, 2019, 2022) for elimination or restriction, and human biomonitoring programs have been implemented (Apel et al., 2020; Brunn et al., 2023) to quantify and follow over time the general population exposure and consequently inform public health actions.

Because chain length is a key determinant of persistence and toxicity (Brunn et al., 2023), long-chain PFAS are being replaced by short-chain alternatives (<C6), initially considered safer. However, studies show that they may be equally persistent and bioactive, with endocrine-disrupting properties and the potential to interfere with brain development (Parsons et al., 2008; Brase et al., 2021; Coperchini et al., 2021). Despite their increasing detection in environmental matrices and human biomonitoring, experimental evidence on the neurodevelopmental toxicity of short-chain PFAS remains scarce.

To address this gap, the present study was designed with a comprehensive experimental approach, combining maternal exposure across multiple critical developmental windows (pre-mating, gestation, and lactation) with behavioral, cellular, and molecular endpoints in young adult offspring. This integrated design allows us to link functional cognitive outcomes with mechanistic alterations in hippocampal development and inflammation. To our knowledge, this is the first study to show that maternal exposure to the short-chain PFASs GenX and perfluorobutanoic acid (PFBA) can impair neurocognitive function and disrupt hippocampal function, thereby providing biologically plausible evidence in support of human epidemiological data and raising urgent concerns about the safety of replacing long-chain PFAS with these compounds.

## 2 Materials and methods

### 2.1 Animals and experimental design

All animal protocols described herein were carried out according to European Community Council Directive 2010/63/EU and Italian legislation (Legislative Decree 26/2014) and in compliance with the Animal Research Reporting of *In Vivo* Experiments (ARRIVE) guidelines and the NIH Guide for the Care and Use of Laboratory Animals. The project has been reviewed by the Animal Welfare Body of the Istituto Zooprofilattico Sperimentale delle Venezie and the IRET Foundation and was approved by the Italian Ministry of Health (authorization no. 206/2022-PR, see Supplementary Material).

Ten 2-month-old CD Sprague–Dawley male rat breeders and twenty-five 2-month-old CD Sprague–Dawley females were included in the study. The contaminated diets were produced (Laboratorio Dottori Piccioni e Mucedola srl., Italy) based on the standard diet (Piccioni, standard pellet feed “Corticella;” Mucedola diet 4RF25) for female rats in the reproduction/lactation phase. Both diets are commercial open formulas, intended for standard maintenance and designed to meet the nutritional requirements of adult mice and rats. Diets were contaminated with either GenX (FRD-902, perfluoroammonium, 2-methyl-3-oxahexanoate, N CAS 62037-80-3, Labochem) or PFBA (HFBA; perfluorobutanoic acid; PFBA, N CAS 375-22-4, Labochem). Contamination was performed for each substance at two intended doses: high dose (H-C) and low dose (L-C). The high dose was intended to be 75 mg/kg in food pellets, with a daily intake of 5 mg/kg body weight. The low dose was intended to be 7.5 mg/kg in food pellets, with a daily intake of 0.5 mg/kg body weight. Doses used in this experiment were based on rat toxicology studies, selecting concentrations for PFBA (Butenhoff et al., 2012) and GenX (Thompson et al., 2019) below the no-observed-adverse-effect level (NOAEL) values. After habituation in the animal facility, the females were randomly divided into five groups:

1. Untreated (standard uncontaminated diet; total offspring: males, n = 16; females, n = 18)
2. L-C GenX: 0.5 mg/kg bw/day (contaminated diet: 7.5 mg GenX/kg food pellets; total offspring: males, n = 15; females, n = 17)
3. H-C GenX: 5 mg/kg bw/day (contaminated diet: 75 mg GenX/kg food pellets; total offspring: males, n = 20; females, n = 21; one pup died before sexing)
4. L-C PFBA: 0.5 mg/kg bw/day (contaminated diet: 7.5 mg PFBA/kg food pellets; total offspring: males, n = 13; females, n = 14; two pups died before sexing)
5. H-C PFBA: 5 mg/kg bw/day (contaminated diet: 75 mg PFBA/kg food pellets; total offspring: males, n = 10; females, n = 13)

Dams were fed for 30 days before mating. Once pregnancy was confirmed through palpation and weight gain observation, each pregnant female was housed individually. Female rats were fed with the experimental diets for approximately 100 days, covering the prenatal and lactation periods. The pups were individually identified, separated from the mother at 5 weeks, and housed in same-sex pairs. All newborn rats were included in the study. Mothers and pups were weighed weekly and clinically monitored for general health conditions. The number of animals per test is shown in the respective figure legends (see the Results section).

Animals were humanely sacrificed at the end of the study period, and samples of plasma, liver, skeletal muscles, kidney, thyroid, and brain were collected and stored at −80 °C for chemical and biomolecular analysis.

### 2.2 Behavioral tests

The test battery was designed taking care to avoid habituation and carryover effects in learning and memory tests and neurobiological substrates (Cnops et al., 2022).

#### 2.2.1 Spontaneous locomotion

Spontaneous locomotion was assessed in a test arena consisting of a 46 cm × 46 cm × 41 cm plastic chamber, with a dark gray floor, virtually divided into 16 fields by ANY-maze Video tracking (ANY-maze, Stoelting-Wood Dale, IL). The light intensity of the room was set at 70 ± 20 Lux before each test session. Rats were individually placed in the center of the chamber, always facing the same direction. Distance traveled and speed over 5 min were measured using ANY-maze trace automatic analysis software. No subjects were excluded from the analysis.

#### 2.2.2 Sensorimotor integration

Rotarod motor coordination and balance were evaluated using a rotarod apparatus (LE 8500 RotaRod: 2Biological Instruments, Varese, Italy). The rotarod test is a 2-day test. Animals were exposed to a single habituation session comprising three trials lasting 5 min in the apparatus on slow fixation velocity (5–10–15 rpm). After 24 h, the rat’s motor ability was evaluated in the test session, during which the rotation speed gradually increased from 4 rpm to 40 rpm over a period of 5 min. We perform three trials, recording the latency to downfall. Due to their large body size, male rats could not be tested, as they exceeded the weight and size limits of the rat-specific equipment. Female rats were tested individually on the apparatus, and latency to fall was automatically recorded for each trial. No female subjects were excluded from the analysis.

#### 2.2.3 Learning and memory

The Morris water maze is the most widely used spatial learning and memory test. Tests were carried out using a tank (diameter 120 cm, height 40 cm) filled with water made opaque by the addition of starch and containing a transparent platform (10 cm diameter). The light intensity of the room was set at 130 ± 20 Lux before each test session, and the water temperature was 20 ± 2 °C. The tank was divided into four equal quadrants by the video tracking software ANY-Maze (ANY-maze software v1, Stoelting Co., Wood Dale, United States), and visual cues were positioned around the pool and on the walls of the room.

The protocol used in this study assessed three cognitive abilities: spatial learning (starting the test from different, random locations around the perimeter of the tank—acquisition phase); reference memory (retention); and reversal learning, to establish whether the animals were able to suppress the information initially learned and adapt their behavior in response to the new position of the platform. Rats were brought into the test room 30 min in advance. On the first day, rats were gently placed for 30 s on the platform positioned 1 cm above the water level and made visible by flags (habituation). The subsequent acquisition phase was carried out with the platform submerged in a fixed position and consisted of three trials per day (with a 1-h interval between trials) for four consecutive days. Rats were placed in the pool facing the wall of the tank, and the latency to reach the platform area (escape latency, acquisition) was recorded. Rats unable to reach the platform within 60 s were gently accompanied onto it for habituation reinforcement. A probe trial was performed 48 h after the final acquisition day (retention), and the latency to reach the platform was recorded. Control group animals that failed to learn to reach the platform within 60 s during the probe trial were excluded from the analysis (one male and one female).

### 2.3 Chemical analysis

Acetonitrile (ACN) (UltraLC-MS grade) and methanol (MeOH) (UltraLC-MS grade) were purchased from VWR Chemicals (Radnor, PA, United States); ammonium acetate (approximately 98%) was purchased from Sigma-Aldrich (St Louis, MO, United States of America). Sodium acetate trihydrate (≥99.5%) and ammonium hydroxide (33% v/v) were supplied by Honeywell Fluka (Charlotte, NC, United States of America). A Milli-Q-Plus ultrapure water system from Millipore (Bedford, MA, United States) was used to prepare Milli-Q water for the preparation of samples and standards. QuEChERS extraction salts (4 g Na_2_SO_4_, 1 g NaCl, 1 g trisodium citrate dehydrate, and 0.5 g disodium hydrogen citrate sesquihydrate) and dispersive SPE 2 mL (150 mg di-primary secondary amine (PSA), 150 mg C18, and 900 mg of MgSO_4_) were supplied by Restek (Bellefonte, PA, United States). A solution of perfluorobutanoic acid (PFBA) and a solution of 2,3,3,3-tetrafluoro-2-(heptafluoropropoxy) propanoic acid (GenX) at concentrations of 2 μg/mL and 50 μg/mL, respectively, were purchased from Wellington Laboratories (Guelph, Canada). Two solutions of labeled standard, perfluoro-n-[^13^C_4_] butanoic acid (MPFBA) and perfluoro-n-[^1,2,3,4,6-13^C_5_] hexanoic acid (M5PFHxA), at a concentration of 2 μg/mL each, were purchased from Wellington Laboratories (Guelph, Canada). Two intermediate mix solutions of PFAS at 10 µg/L and 100 μg/L, respectively, were prepared by mixing the appropriate amounts of the corresponding solutions and diluting with HPLC-grade methanol.

An intermediate mix solution of the labeled compounds, each at a concentration 10 μg/L, was prepared by appropriate dilution with HPLC-grade methanol of the pristine two solutions (Wellington Laboratories). Six standard solutions of PFBA and GenX, ranging from 0.125 µg/L to 7.5 μg/L (corresponding to 0.025–1.5 μg/kg in the sample), were prepared for quantification.

For chemical analysis of tissues, 0.5 g of homogenized matrix (liver, kidney, brain, and thyroid) was weighed in a polypropylene conical centrifuge tube, fortified with 5 μL of mass-labeled PFAS mixture at 10 ng/mL, mixed with 2 mL of Milli-Q Water (MQW), and shaken on a vortex-mixer (IKA Vibrax VXR, Staufen, Germany) for 1 min. After the addition of 2.5 mL of acetonitrile, samples were further shaken on an automatic stirrer (Genogrinder™, Spex® Sample PREP, Stanmore, United Kingdom) at 25 Hz for 3 min. Afterward, 1.30 g of QuEChERS extraction salts were added. Samples were shaken again on an automatic stirrer at 25 Hz for 1 min and centrifuged for 5 min at 6,000× g (Eppendorf Centrifuge 5810, Amburg, Germany). All the extracts were submitted to d-SPE clean-up. In detail, 1 mL aliquots of the extracts were transferred into a 2 mL plastic tube containing PSA, C18, and MgSO_4_, shaken on an automatic stirrer at 25 Hz for 3 min, and then centrifuged for 10 min at 6,000× g. Finally, 600 µL of extract was evaporated until dry under a gentle stream of N_2_ at 40 °C. The dried residue was dissolved in 120 µL of 10 mM ammonium acetate in 80% MeOH and 20% ACN. The final solution was transferred into vials for analysis by liquid chromatography-tandem mass spectrometry (LC-MS/MS).

LC-MS/MS analysis was performed on a Shimadzu HPLC system (Kyoto, Japan) coupled to an API 65 AB SCIEX (Framingham, MA, United States) Triple Quadrupole (QQQ).

The sample (5 μL) was injected into a Waters Aquity UPLC BEH Shield RP 18 (2.1 mm × 100 mm), 1.7 μm column.

The eluents consisted of water with 10 mM ammonium acetate (FM A) and MeOH/ACN (80/20) with 10 mM ammonium acetate (FM B). The gradient applied was: 0–0.1 min of 5% FM B, then FM B was increased linearly to reach 95% FM B at 8 min and kept for 2 minutes. Finally, the concentration of FM B was decreased to 5% between 10 min and 10.5 min, and the column was equilibrated to initial conditions until 14 min. The flow rate was 0.3 mL/min, and the column oven temperature was set at 30 °C.

The MS/MS instrument was operated in negative electrospray ionization (ESI) mode. The gas temperature and the ion spray voltage were kept at 350 °C and −2500 V, respectively.

Ions were monitored using a multiple reaction monitoring (MRM) mode. Transitions for each target analyte are reported in Supplementary Table S1.

The isotope dilution method was used to analyze the samples. In particular, the corresponding labeled compounds for PFBA and M5PFHxA for GenX were used as internal standards to calculate the relative response factor of the corresponding native compound and to confirm the retention time (RT).

Analyst software AB SCIEX (Framingham, MA, United States) was used to control the LC-MS system, and Multiquant 3.0.2 software (Framingham, MA, United States) was used to quantify analytes.

Analytical method validation and quality control are described in detail in the Supplementary Material.

See Supplementary Table S1 for instrumental mass spectrometry settings and Supplementary Table S2 for the analytical method recovery and repeatability measurements. See Supplementary Tables S3–S7 for a detailed list of samples and pooled samples submitted to chemical analysis.

### 2.4 Hormonal assays

Hormones were measured in plasma samples, obtained after centrifugation of blood collected in tubes with K3-EDTA anticoagulant, at 1730 × g for 15 min at 4 °C. The Cobas e402 analyzer (Roche Diagnostics, Mannheim, Germany) and commercial diagnostic kits from Roche Diagnostics were used. The analytical characteristics of each method are reported in Supplementary Table S8.

### 2.5 Semi-quantitative polymerase chain reaction (PCR) analysis and qPCR pathway arrays

Gene expression analysis was performed in the hippocampus at the time of sacrifice. The animals used for this analysis were: male ctrl, n = 6; male PFBA H-C, n = 6; male GenX H-C, n = 6; female ctrl, n = 4; female PFBA H-C, n = 3; female GenX H-C, n = 6. The total RNA was extracted from the homogenized tissues using the RNeasy Plus Universal Mini Kit (Qiagen, Hilden, Germany) following the manufacturer’s instructions and quantified by a NanoDrop 2000 spectrophotometer (Thermo Fisher Scientific. The iScript™ cDNA Synthesis Kit (Bio-Rad, Hercules, CA, United States) was used for cDNA synthesis, following the manufacturer’s instructions, with 1 µg (1,000 ng) of RNA from each rat. A no-reverse transcription sample was added, using the iScript Supermix No-RT Control instead of the reverse transcription enzyme, and processed for gene expression analysis in parallel with the other samples to check for genomic DNA contamination. The qPCR reaction was performed using the CFX96 machine (Bio-Rad) and the SsoAdvanced™ Universal SYBR® Green Supermix (Bio-Rad), starting with 10 ng of cDNA. Relative mRNA quantification was obtained using the comparative cycle threshold (Cq) method. Cq values were collected for each sample and normalized to the housekeeping gene glyceraldehyde 3-phosphate dehydrogenase (GAPDH). Specific primers were used to analyze the expression of the target genes (see Supplementary Table S4), with a tested efficiency of approximately 2. The 2^−(ΔΔCq)^ method was used as a semi-quantitative method, initially normalizing the Cq of the target gene with the housekeeping gene expression (ΔCq) and subsequently with a control group (ΔΔCq), as specified in the figure legend for each graph. The relative expression is then shown as a fold change relative to the control group.

For pathways qPCR array analysis, the PARN-125Z and PARN-126Z plates (Qiagen) were used to analyze differentially expressed genes (DEGs) in male and female animals exposed to GenX and PFBA H-C compared to control animals. The cDNA was prepared using the RT2 First Strand kit (Qiagen), using a pool of n samples for each group (1 µg per pool): male ctrl (n = 6), male PFBA H-C (n = 6), male GenX H-C (n = 6), female ctrl (n = 4), female PFBA H-C (n = 3), female GenX H-C (n = 6). The RT2 Profiler SYBR Green Mastermix (Qiagen) was used for the reaction. The temperature cycle profile was the standard requested by the manufacturer: Polymerase activation (95 °C, 10 min), 1 cycle; Amplification (95 °C, 15 s; 60 °C, 1 min), 40 cycles; melting curve.

For data analysis, the same baseline threshold was selected for all the plates. Then, Cq data were exported and uploaded into Gene Globe online software (Qiagen) for the analysis. For the PARN-125Z array, the *B2M* gene was selected as housekeeping among the five housekeeping genes included in the plates; for the PARN-126Z array, the average value of all five housekeeping genes was used (*RPLP1*, *LDHA*, *ACTB*, *B2M*, and *HPRT1*). Each qPCR array contains quality controls for reproducibility, a positive PCR control, and genomic DNA contamination. All the controls resulted in “passed” for all the samples. The “fold of change” data were used to compare the treated animal groups with the control animals, within the same sex.

STRING online software (https://string-db.org/) was used to analyze the functional protein association network of the protein encoded by the identified DEGs and the related pathway enrichment analysis using the Gene Ontology database for biological process and molecular function. For the STRING analysis, the following active interaction sources were used: textmining, experiments, databases, and co-expression. The connection between nodes is based on “evidence,” and the network type selected was “full STRING network,” using a minimum required interaction score with a medium confidence equal to 0.400. For the clusterization, the k-means clustering with a defined number of clusters = 3, as automatically suggested by the software based on the number of nodes, was used.

### 2.6 Histological and immunofluorescence analysis

Animals were sacrificed by isoflurane (5%) overdose and transcardially perfused with 10 mL of 0.1 M PBS followed by 80 mL of ice-cold 4% paraformaldehyde and 14% picric acid in 0.2 M Sorensen buffer overnight. Then, the tissues were washed in 0.2 M Sorensen buffer containing 5% sucrose at least five times and frozen with liquid N_2_. Coronal cryostat sections (Leica CM1950 Biosystems, Walldorf, Germany) of the dorsal hippocampus, 14 µm thick, were collected and processed for indirect immunofluorescence using anti-doublecortin (Rat, 1:800, BD Pharmingen, cat 553792, clone 1D4B, San Diego, CA, United States) and secondary antibodies conjugated with FITC (Jackson ImmunoResearch, Cambridgeshire, United Kingdom). A Nikon Ni equipped with the Nikon camera DS Qi2 was used for imaging. The overall brightness and contrast of the whole image were lightly adjusted to allow background visualization using Microsoft PowerPoint software. The same corrections were applied in all images included in Figure 5.

### 2.7 Cell culture, immunocytochemistry, and image analysis

Hippocampal neurons were derived at birth from Sprague–Dawley rat pups born to mothers fed PFBA-contaminated feed (5 mg/kg/p.c./day) or standard diet, for a period covering 1 month before mating and 21 days of gestation. For each experimental group, all the pups born from two mothers were used, for a total of 12 pups for each group. Hippocampal neurons derived from pups of the same experimental group were pooled, and five technical replicates for each group were analyzed. In brief, the animals were decapitated. The heads were placed in a Petri dish containing 1× PBS with 1% penicillin/streptomycin (100 U × mL^−1^/100 μg × mL^−1^). Using the stereomicroscope and forceps, the meninges and cerebellum were removed. Next, an incision along the midline of the brain separated the two hemispheres, exposing the hippocampi. The hippocampi were then dissected and placed in 1.5 mL tubes containing a non-enzymatic dissociation buffer (Sigma-Aldrich, St. Louis, MO, United States). After 15 min of incubation at 37 °C, mechanical dissociation was performed by several pipettings. Following 5-min centrifugation at 400 × g, the cell pellets were resuspended in neurobasal culture medium supplemented with 1× B27 (Thermo Fisher Scientific), 2 mM glutamine (Sigma-Aldrich), and 1% penicillin/streptomycin (100 U × mL^−1^/100 μg × mL^−1^) (Thermo Fisher Scientific) and then seeded in 96-well plates coated with Cultrex 2D substrate (0.25 mg/mL, Trevingen). The cells were kept in a humidified incubator at 37 °C with 5% CO_2_, and half of the medium was changed every 3 days.

Cultures were analyzed at specific time points (7 and 21 DIV, days *in vitro*) using immunocytochemistry coupled with high content screening technology (Cell Insight™ CX5, Thermo Fisher Scientific), which allows automated acquisition and analysis of the entire culture (>5,000 cells/well). At the established time point, cells were fixed with cold 4% paraformaldehyde for 20 min at room temperature. Following a quick wash in PBS, the cells were subsequently incubated for 1 h with BSA 5%, in PBS 0.3% Triton-X 100. After this, they were left overnight with the primary antibody. The following day, they were incubated with the secondary antibody. Several antibodies were used, including anti-β-III-tubulin (Ms, 1:500, Santa Cruz Biotechnology, Dallas, TX, United States) and anti-MAP2, microtubule-associated protein 2 (Rb, 1: 250, Santa Cruz Biotechnology) as markers of neural maturation; anti- PSD95, postsynaptic density protein 95, (Rb, 1:500, Abcam, Cambridge, United Kingdom); anti-VGLU1, glutamate vesicular transporter 1 (Rb, 1:300, Abcam) and anti-VGAT, GABA vesicular transporter 1 (Rb, 1:500, Millipore, Merck KGaA, Darmstadt, Germania) as synaptic markers to quantify synaptic maturation. Cy2-conjugated anti-mouse (1:500, Jackson Laboratories, Bar Harbor, ME, United States) and RRX-conjugated anti-rabbit (1:500, The Jackson Laboratory) were used as secondary antibodies. Cells were also incubated with DAPI nuclear dye (1 μg/mL in PBS, 0.3% Triton-X 100) to detect nuclei.

Based on b-III-tubulin and MAP2 staining, the high-content screening (HCS) technology was used to quantify the neuronal maturation. The HCS machine (Cell Insight XT, Thermo Fisher Scientific) and the software (HCS Studio v 6.6.0) were used for the image acquisition and analysis. The “Neuronal profiling” algorithm was used to identify each cell body (using the nuclear staining and the marker staining) and to trace all the neurites. By combining the neuronal identification and the neurite tracing elements, it was possible to measure the following readouts: neurite total count, total and average length, and ramification index.

Confocal microscopy has been used to study synaptic maturation. Slides were analyzed with a Nikon Ti-E fluorescence microscope connected to an A1R confocal system (Nikon, Minato, Tokyo, Japan). Images were acquired with a ×40 objective at 1,024 × 1,024 resolution, and all z-stacks were collected in accordance with the optical section separation values (z-Interval) suggested by the NIS-Elements AR 3.2 software. IMARIS software (v.9.7; Andor Technology Limited, Belfast, United Kingdom) was used to process the confocal 3-D stacks. Using nuclear staining, each nucleus, corresponding to each cell, was identified. The “spot” algorithm was used to identify the synaptic markers around each cell, counting the number of spots per cell.

### 2.8 Statistical analysis

The statistical analyses and graphs were performed using GraphPad Prism v10.2.2 (GraphPad Software, San Diego, CA, United States). All data are presented as individual values and mean ± standard error of the mean (SEM). Data have been analyzed for normality using the Kolmogorov–Smirnov test.

For normally distributed data, Student’s t-test was used to compare the means between two groups, while one-way and two-way ANOVA and post hoc Dunnett’s multiple comparisons tests were used to compare the means between three or more groups.

For non-normal data, the Mann–Whitney test was used to compare the means between two groups, while the Kruskal–Wallis and post hoc Dunn’s multiple comparisons tests were used to compare the means between three or more groups.

For statistical analysis of PFBA and GenX residues in tissues, data from female and male offspring of the same group were merged to report the mean, standard deviation, median, and median absolute deviation (MAD) from the median according to each animal group. The lower bound approach was used (zero value was assigned to results < LOQ). R software version 4.2.3 was used for boxplot data visualization.

The analysis performed in each experiment is described in the Results section, where p-values related to the overall test are reported, and in the respective figure legends. The number of animals included in each test is reported in the figure legends. Results were considered significant when the probability of their occurrence due to chance alone was less than 5% (p < 0.05).

## 3 Results

### 3.1 Experimental schedule and animal monitoring

**FIGURE 1:**
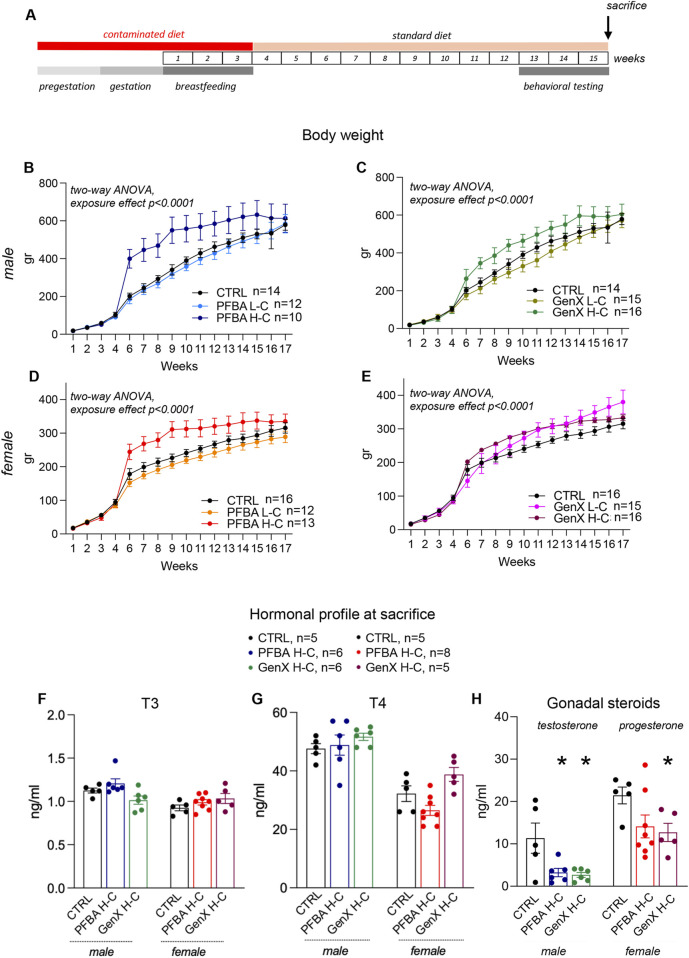
Experimental design, rat body weight growth, and hormonal profile at sacrifice. **(A)** Timeline of the experimentation. Mothers were fed with a contaminated diet prior to mating, during gestation, and during lactation. Pups were fed with a standard diet and tested in adulthood for complex behaviors: spontaneous locomotion, sensory-motor integration, learning, and memory. **(B–E)** Body weight growth of male **(B**,**C)** and female **(D**,**E)** offspring from PFBA **(B**,**D)** and GenX **(C**,**E)** exposed mothers. A significant treatment-induced effect was observed compared to control groups (two-way ANOVA, p < 0.0001), showing that in H-C treatment groups, maternal exposure to contaminants induces a higher body weight gain. **(F–H)** Hormonal profile of offspring at sacrifice. T3 **(F)**, T4 **(G),** and gonadal steroids **(H)** were measured. Statistical analysis: unpaired t-test, *p < 0.05; **p < 0.01; ****p < 0.0001. The number of animals in each group is indicated in the figure legend.

**FIGURE 2:**
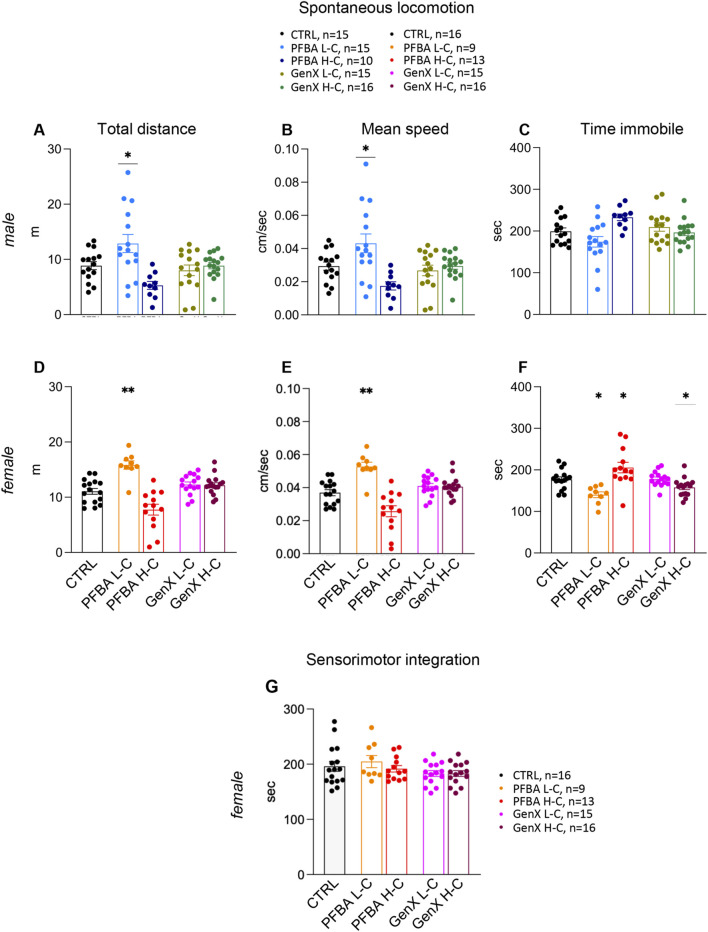
Spontaneous locomotion and sensorimotor integration. **(A–F)** Spontaneous locomotion of both sexes in an open-field arena expressed as distance traveled **(A**,**D)**, mean speed **(B**,**E)**, and time immobile **(C**,**F)**. Results are expressed as individual values and mean +SEM. Statistical analysis: one-way ANOVA and Dunnett’s post hoc test, *p < 0.05; **p < 0.01. **(G)** The rotarod test in female rats indicated the latency to fall. Results are expressed as individual values and mean +SEM. Statistical one-way ANOVA, followed by Dunnett’s post hoc test. The number of animals in each group is indicated in the figure legend.

The GenX and PFBA exposure schedule and the behavioral test battery are shown in Figure 2A. Mothers were exposed to contaminated diets until weaning. Then, the pups were fed with a standard diet for the rest of the experiments. Tests were performed starting from 65 days after weaning, and offspring were sacrificed from 52 days to 85 days after weaning. No mortality and no gross health problems were observed among either the mothers or the litters in the experimental and control groups. Offspring weight gain of male (Figures 1B,C) and female (Figures 1D,E) rats generated from GenX and PFBA-exposed dams (both high and low concentrations) indicate a trend toward increased weight gain in juvenile rat born from high-dose contaminant exposure (statistical analysis: PFBA offspring male: two-way ANOVA, time p < 0.0001, F (2.629, 86.75) = 2,397; treatment p < 0.0001, F (2, 33) = 44.27, interaction p < 0.0001, F (30, 495) = 41.35; GenX offspring males: two-way ANOVA, time p < 0.0001, F (3.545, 148.4) = 3,153; treatment p < 0.0001, F (2, 42) = 47.38, interaction p < 0.0001, F (30, 638) = 24.81; PFBA offspring female: two-way ANOVA, time p < 0.0001, F (2.947, 112.0) = 5028; treatment p < 0.0001, F (2, 38) = 75.71, interaction p < 0.0001, F (30, 570) = 64.58; GenX offspring female two-way ANOVA, time p < 0.0001, F (3.102, 136.3) = 3,275; treatment p < 0.0001, F (2, 44) = 13.97, interaction p < 0.0001, F (30, 659) = 28.68).

Hormonal plasma levels at sacrifice (e.g., 12 weeks after PFBA and GenX discontinuation) are reported in Figures 1F–H. While slight changes are observed in the thyroid profile, specifically a slight trend toward an increase in T4 in the male PFBA-treated litters, a substantial decline was found in the testosterone blood level in both GenX- (Student’s t-test, p = 0.0409) and PFBA-exposed male rats (Student’s t-test, p = 0.0268) and in progesterone in GenX-exposed female rats (Student’s t-test, p = 0.0174).

### 3.2 Residual analysis in food pellets and tissues

The residual concentration of GenX and PFBA in the food pellets used in the *in vivo* experiments is reported in Table 1. While PFBA-contaminated diets substantially reflect the estimated concentrations, GenX contamination resulted in lower concentrations than expected. In the text and figures, the two dosages are indicated as “low concentration” (L-C) and “high concentration” (H-C), respectively. The diet produced by Piccioni was used for behavioral studies, while the diet produced by Mucedola was used for *ex vivo* cell culture experiments.

**TABLE 1:** GenX and PFBA concentrations (mean ± SD) in food samples.

Diet	Treatment	mg/kg food pellets
GenX-contaminated diet by Piccioni	Low concentration (L-C)	2.46 ± 0.58
	High concentration (H-C)	12.73 ± 5.91
PFBA-contaminated diet by Piccioni	Low concentration (L-C)	4.49 ± 0.36
	High concentration (H-C)	59.57 ± 0.51
PFBA-contaminated diet by Mucedola	High concentration (H-C)	42.47 ± 4.44

Diet	PFAS analysed	mg/kg food pellets
Control diet by Mucedola	PFBA	0.00080 ± 0.00007
	GenX	0.00065 ± 0.00009

The means of merged data of GenX and PFBA residues in tissues of different experimental groups at sacrifice (12 weeks after weaning) are reported in Table 2. All animals eating the control diet did not show PFBA and GenX residue accumulation (< LOQ = 0.05 and 0.10 ng/g, respectively) in all the tissues investigated (see repository). In GenX-contaminated diets, residues were not detected in tissues of offspring (see repository). In PFBA-contaminated diets, residues were detected in several livers and kidneys of male and female offspring from the L-C diet group, even though some groups had residue values < LOQ = 0.05 ng/g. In groups receiving the H-C diet, PFBA was detected (>LOQ = 0.05 ng/g) in all livers and kidneys of litters. In the brain, PFBA residues were detected at low doses in some groups receiving the H-C diet, while no residues were detected in the L-C groups (see repository).

**TABLE 2:** GenX and PFBA residues in livers, kidneys, and brains of litters (means of merged data ± SD), 12 weeks after weaning.

Treatment	Animal	N	Liver	Kidney	Brain
			ng/g of tissue ± SD		
Controls	Litter, female	4	N.D.	N.D.	N.D.
	Litter, male	2	N.D.	N.D.	N.D.
GenX, L-C	Litter, female	3	N.D.	N.D.	N.D.
	Litter, male	3	N.D.	N.D.	N.D.
GenX, H-C	Litter, female	3	N.D.	N.D.	N.D.
	Litter, male	3	N.D.	N.D.	N.D.
PFBA, L-C	Litter, female	3	0.047 ± 0.042	0.017 ± 0.029	N.D.
	Litter, male	2	0.060 ± 0.049	0.035 ± 0.041	N.D.
PFBA, H-C	Litter, female	2	0.125 ± 0.035	0.255 ± 0.148	0.030 ± 0.042
	Litter, male	2	0.525 ± 0.361	0.110 ± 0.014	0.040 ± 0.057

Abbreviation: N.D., not detected.

### 3.3 Short-chain PFAS exposure during gestation and breastfeeding alters spontaneous locomotion, learning, and memory in adult rats

All pups of both sexes were tested in adulthood (from 13 to 16 weeks of age) for complex behaviors (spontaneous locomotion, sensorimotor integration, learning, and memory using the Morris water maze), and the number of rats included in each test is reported in the figures.

Spontaneous locomotion was evaluated as a 5-min spontaneous exploration of a novel, non-stressful environment. Distance traveled (A male and D female), mean speed (B male and E female), and time immobile (C male and F female) are reported in Figure 2. No effect of GenX exposure was observed in male rats. Significantly higher activity was observed in the PFBA low-concentration diet group for males (distance traveled, one-way ANOVA, p = 0.0006, F(9,102), CTRL vs. PFBA L-C Dunnett’s post hoc test, p = 0.0328; mean speed, one-way ANOVA, p = 0.0008, F (8,782), CTRL vs. PFBA L-C Dunnett’s post hoc, p = 0.0339). A similar trend was observed in female rats, with higher activity in the PFBA low concentration group (distance traveled, Kruskal–Wallis test, p < 0.0001, CTRL vs. PFBA L-C Dunnett’s post hoc test, p = 0.0079; mean speed, Kruskal–Wallis test, p < 0.0001, CTRL vs. PFBA L-C Dunnett’s post hoc test, p = 0.0072). In addition, a different time immobile was observed in female rats in all treated groups (one-way ANOVA, p = 0.0003, F(10,57), CTRL vs. PFBA L-C Dunnett’s post hoc test, p = 0.0205; CTRL vs. PFBA H-C Dunnett’s post hoc test, p = 0.0488; and one-way ANOVA, p = 0.0160, F (4,550), CTRL vs. GenX H-C Dunnett’s post hoc test, p = 0.0265).

The sensory-motor integration, as assessed by rotarod performance, did not differ significantly among control, low-dose, and high-dose PFAS-exposed groups, indicating that perinatal PFAS exposure does not seem to impair this functional domain in offspring (Figure 2G).

**FIGURE 3:**
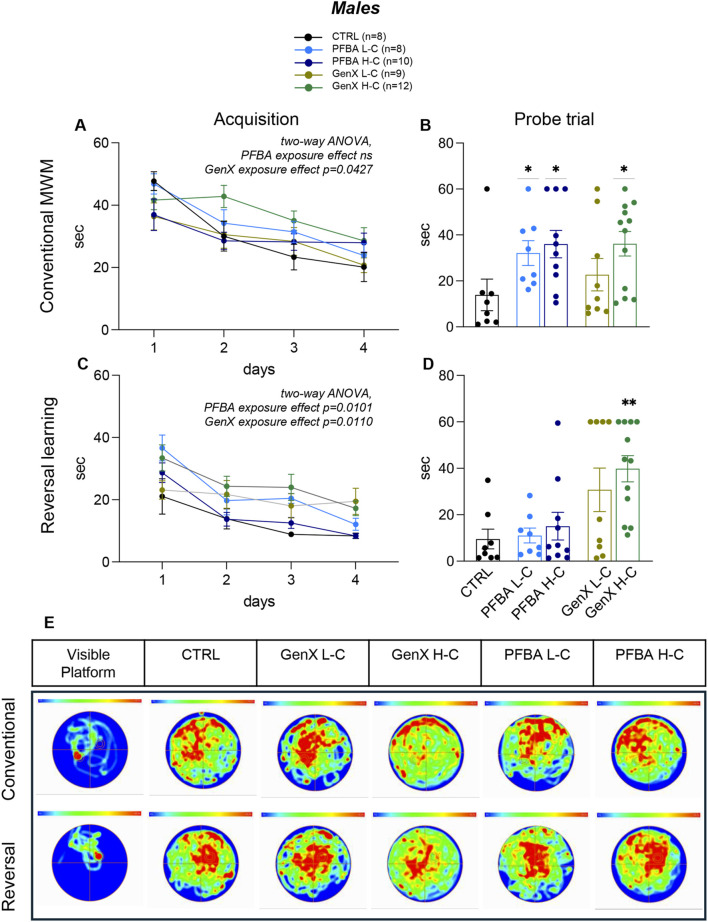
Spatial learning and memory in males. **(A**,**C)** Graphs show the acquisition phase in the conventional Morris water maze paradigm **(A)** and in the reversal learning paradigm **(C)**, where the latency to reach the platform over four consecutive days is reported. **(B**,**D)** The graph bars show the latency to the first entry in the platform zone in the probe trial in the conventional **(B)** and the reverse learning phase **(D)**. Image **(E)** represents the average heat map of the animals’ center points for each experimental group, for the time interval 0–60 s, using a color scale from 0 to 0.3 s (hottest color). The top row shows the analysis during the probe trial at the end of the acquisition phase, while the bottom row shows the corresponding analysis during the probe trial of the reversal learning phase. The “visible platform” images depict the control test. Data are expressed as mean ± SEM. The number of rats in each group is reported in the legend. Statistical analysis: acquisition phase, two-way ANOVA (see Results for details); probe test, one-way ANOVA and post hoc Dunn’s test, *p < 0.05; **p < 0.01.

**FIGURE 4:**
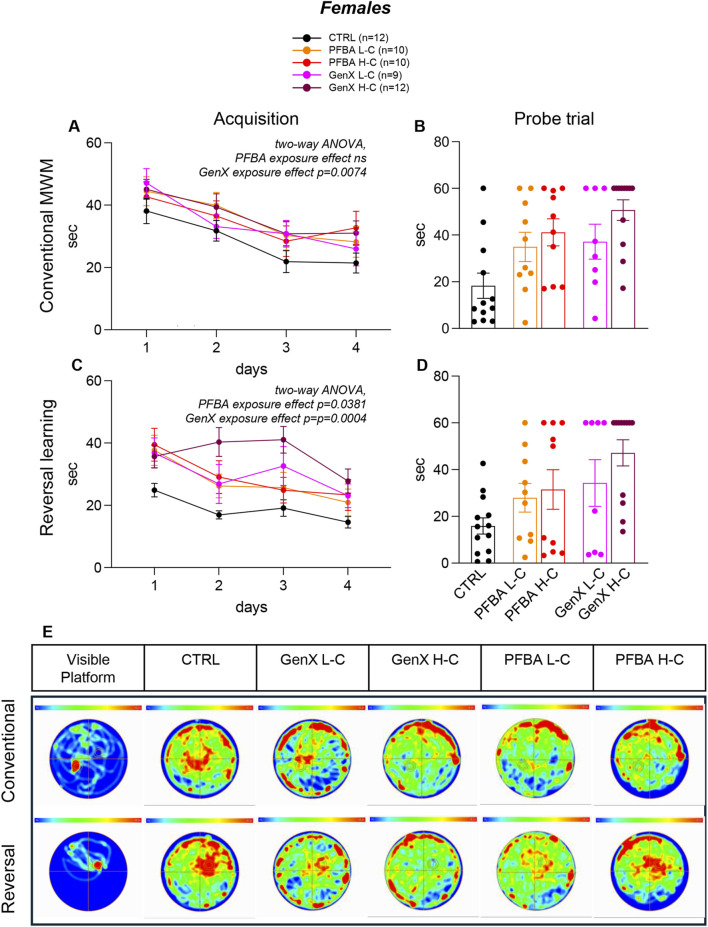
Spatial learning and memory in females. **(A**,**C)** Graphs show the acquisition phase in the conventional Morris water maze paradigm **(A)** and in the reversal learning paradigm **(C)**, where the latency to reach the platform over four consecutive days is reported. **(B**,**D)** The graph bars show the latency to the first entry in the platform zone in the probe trial in conventional **(B)** and reverse learning phase **(D)**. Image **(E)** represents the average heat map of the animals’ center points for each experimental group, for the time interval 0–60 s, using a color scale from 0 to 0.3 s (hottest color). The top row shows the analysis during the probe trial at the end of the acquisition phase, while the bottom row shows the corresponding analysis during the probe trial of the reversal learning phase. The “visible platform” images depict the control test. Data are expressed as mean ± SEM. The number of rats in each group is reported in the legend. Statistical analysis: acquisition phase, two-way ANOVA (see Results for details); probe test, one-way ANOVA and *post hoc* Dunn’s test, *p < 0.05; **p < 0.01.

The Morris water maze test was used to assess cognitive abilities, spatial learning during the acquisition phase, reference memory *via* a probe trial, and cognitive flexibility during the reversal learning phase. During the acquisition phase, a time-dependent effect (learning) was observed, but no treatment effect was detected in the PFBA groups (two-way, males: time p < 0.0001, F(2.469, 56.78) = 19.72; treatment ns; interaction ns.; females: time p < 0.0001, F(2.698, 78.25) = 10.09; treatment ns; interaction ns; Figures 3A, 4A), whereas a treatment effect was evident in the GenX group (two-way ANOVA, male: time p < 0.0001, F(2.697, 70.13) = 15.05; treatment p = 0.0427, F(2, 26) = 3.570; interaction ns; females: time p < 0.0001, F(3, 120) = 11.82; treatment p = 0.0074, F(2, 120) = 5.114; interaction ns, Figures 3A, 4A).

Male and female rats treated with either PFBA and GenX exhibited significant impairments in reversal learning phase (two-way ANOVA, PFBA: males, time p < 0.0001, F(2.009, 46.22) = 33.04; treatment p = 0.0101, F(2, 23) = 5.644; interaction ns.; females, time p < 0.0001, F(2.538, 73.61) = 14.61; treatment p = 0.0381, F(2, 29) = 3.665; interaction ns; GenX: males, time p = 0.0007, F(2.734, 71.08) = 6.748; treatment p = 0.0110, F(2, 26) = 5.395; interaction ns; females, time p < 0.0004, F(2.618, 78.53) = 7.405; treatment p = 0.0004, F(2, 30) = 10.22; interaction ns, Figures 3C, 4C).

In the probe trials, significant differences were observed in males exposed to PFBA at low and high concentrations, as well as GenX at high concentrations, compared to controls (Kruskal–Wallis test, p = 0.0174, Dunn’s post hoc test: CTRL vs. PFBA L-C, p = 0.0380; CTRL vs. PFBA H-C, p = 0.0183; Kruskal–Wallis test, p = 0.0432, Dunn’s post hoc test: CTRL vs. GenX H-C, p = 0.03641; Figure 3B). In females, both high-concentration exposure groups exhibited significant impairments relative to controls (Kruskal–Wallis test, p = 0.0238, Dunn’s post hoc test: CTRL vs. PFBA H-C, p = 0.0151; Kruskal–Wallis test, p = 0.0017, Dunn’s post hoc test: CTRL vs. GenX H-C, p = 0.0008, Figure 4B). The probe trial of reversal-phase confirmed the detrimental effects of high-concentration GenX treatment in both sexes compared to controls, with significant impairments observed in latency to locate the platform in males (Kruskal–Wallis test, p = 0.0135, Dunn’s post hoc test: CTRL vs. GenX H-C, p = 0.0069, Figure 3D) and females (Kruskal–Wallis test, p = 0.0052, Dunn’s post hoc test: CTRL vs. GenX H-C, p = 0.0024, Figure 4D).

In panels E of Figure 3 (male) and Figure 4 (female), the heat maps of probe trials visually depict the same data in the form of a map in which data values are represented as colors, where red represents the most visited areas in the pool. The control “flag trial” performed using the visible platform is presented in the “visible platform” panel. This trial, performed immediately after the probe test, confirmed that all animals could correctly see and reach the platform when it was visible. In both male (Figure 3E) and female (Figure 4E), the heat maps confirmed, by a clear visual complement to the quantitative data of the probe test, the predominantly detrimental impact of both GenX and PFBA on spatial memory retention, especially at higher concentrations. Distinct exploration patterns emerge from different groups. Quantitatively, the thigmotaxis analysis revealed significant differences only in females exposed to PFBA and GenX at low concentrations compared to controls (Kruskal–Wallis test, p = 0.0174; Dunn’s post hoc test: CTRL vs. PFBA L-C, p = 0.0084; CTRL vs. GenX L-C, p = 0.0402; see Supplementary Figure S1). No differences were detected in males.

### 3.4 Short-chain PFAS exposure during gestation and breastfeeding impairs neurogenesis: *ex vivo* tissue analysis and cell culture

To test the hypothesis of a possible impact of prenatal exposure to short-chain PFAS on hippocampal developmental neurogenesis, we performed two sets of experiments.

**FIGURE 5:**
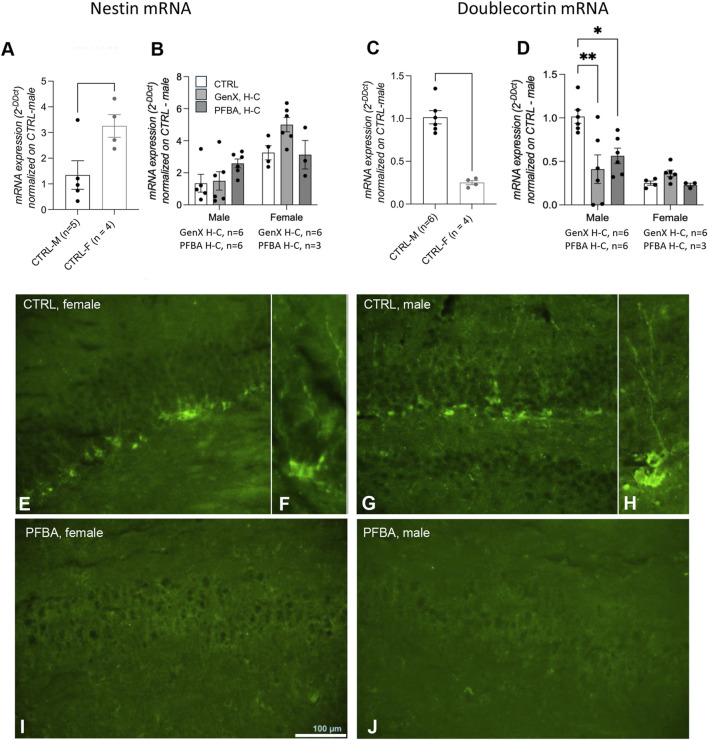
Hippocampal neurogenesis in adult offspring born from control and exposed mothers. Nestin **(A**,**B)** and doublecortin **(C**,**D)** mRNA expression levels in the hippocampus in male vs. female control rats **(A**,**C)**. Data are expressed as mean ± SEM, and individual values are also shown. Statistical analysis: Unpaired t-test between the indicated groups **(A**,**C)**; one-way ANOVA followed by Dunnett’s post hoc **(B**,**D)** (*p < 0.05; **p < 0.01; ****p < 0.0001). **(E–J)** Representative micrographs of doublecortin immunoreactivity in the dentate gyrus in control female **(E**,**F)** and male rats **(G**,**H)**, and offspring from PFBA H-C-exposed mothers **(I)** female; **(J)** male. The number of animals in each group is indicated in the figure legend.

We first investigated neurogenesis in the dentate gyrus of the hippocampus in adult rats by gene and protein expression analysis of nestin, a marker of neuroepithelial stem cells (Jain et al., 2019), and doublecortin, a marker of proliferating neuroblasts (Yagi et al., 2024). Nestin gene expression in control rats was higher in female than in male rats (Figure 5A, Student’s t-test, p = 0.0364), and no differences were found in exposed male and female rats (Figure 5B). In addition, doublecortin mRNA expression was higher in male than female rats (Figure 5C, Student’s t-test, p < 0.0001). The exposure to both GenX and PFBA at high concentration produced a significant reduction in males (Figure 5D, one-way ANOVA, p = 0.0063 F (7.240) and post hoc Dunnett’s CTRL vs. PFBA H-C, p = 0.0283, CTRL vs. GenX H-C, p = 0.0044), but not in females. Immunofluorescence of doublecortin-positive neuroblasts in the dentate gyrus of the hippocampus confirmed the substantial absence of neuroblasts in the hippocampus of PFBA-exposed rats (Figures 5E–J).

**FIGURE 6:**
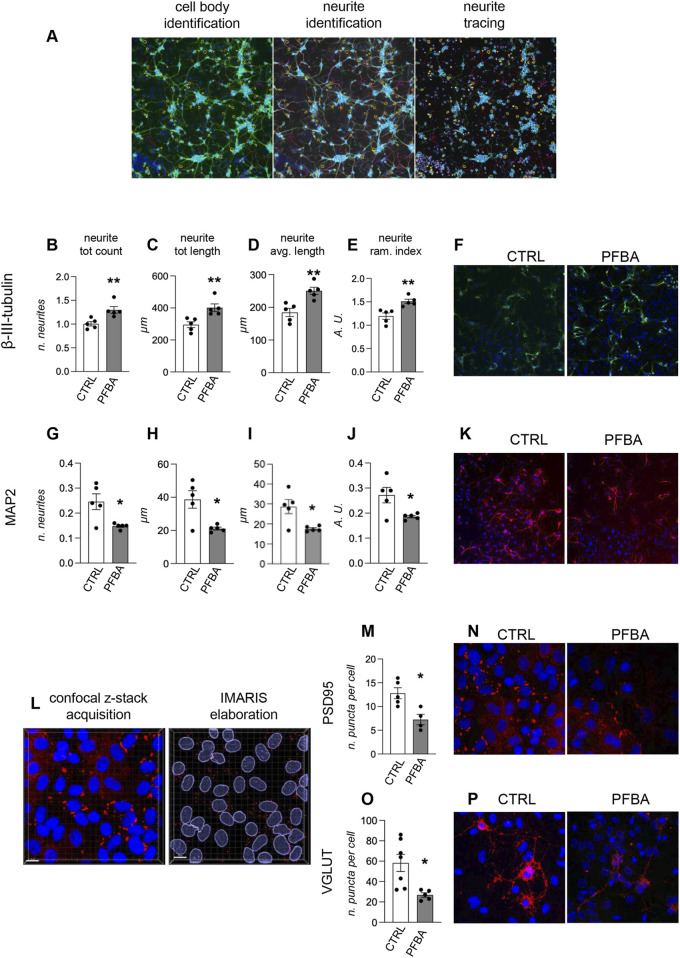
*Ex vivo* analysis of primary neuronal hippocampal cultures. **(A)** Cell-based high content screening analysis of neuronal maturation through neurite elongation study. The “neuronal profiling” algorithm was used for the analysis. The protocol includes identifying the cell bodies using nuclear staining and the neuronal marker. Using the same marker, the software identifies the neurites emanating from each cell body and reconstructs the neurite net. **(B–E)** Quantification of the neurite elongation using beta-III-tubulin staining and four different parameters: neurite total count per neuron **(B)**, neurite total length per neuron **(C)**, neurite average length per neuron **(D)**, and neurite ramification index **(E)**. Technical replicates for each experimental group: n = 5. **(F)** Representative images of primary hippocampal cultures isolated from the control group (CTRL) and animals exposed to PFBA, stained for beta-III-tubulin. **(G–J)** Quantification of the neurite elongation using beta-III-tubulin staining and four different parameters: neurite total count per neuron **(G)**, neurite total length per neuron **(H)**, neurite average length per neuron **(I)**, and neurite ramification index **(J)**. Technical replicates for each experimental group: n = 5. **(K)** Representative images of primary hippocampal cultures isolated from the control group (ctrl) and animals exposed to PFBA, stained for MAP2. **(L)** The synaptic markers were analyzed using confocal imaging and voxel-based analysis using IMARIS software. Nuclear staining (DAPI) was used to identify the cells, and using the specific synaptic marker, the puncta were identified using the “spot” algorithm of the software. **(M)** Quantification of the number of PSD95-positive spots per cell (technical replicates: CTRL n = 5, PFBA n = 4). **(N)** Representative images of primary hippocampal cultures isolated from the control group (ctrl) and animals exposed to PFBA, stained for PSD95. **(O)** Quantification of the number of VGLUT-positive spots per cell (technical replicates: CTRL n = 7, PFBA n = 5). **(P)** Representative images of primary hippocampal cultures isolated from the control group (ctrl) and animals exposed to PFBA, stained for VGLUT. Data are expressed as mean ± SEM and technical replicates are represented as dots. Statistical analysis: Unpaired t-test (*p < 0.05; **p < 0.01).

We then isolated primary hippocampal neurons from newborn rats (within 24 h from birth) after PFBA H-C gestational exposure of the mothers. Cultures were analyzed at 7 DIV, corresponding to the start of neurite net maturation, and at 21 DIV, corresponding to a fully mature system (Jaberi et al., 2021). At 7 DIV, cultures were stained for a pan-neuronal marker (beta-III-tubulin) and a marker of the mature neurons (MAP2). The analysis was performed using the neurite identification algorithm of the cell-based HCS to detect and quantify the whole neuronal network in the whole culture (Figure 6A). Using the beta-III-tubulin marker, the cultures isolated from the PFBA-exposed rats were more developed. The neurite total count per neuron was higher (Student’s t-test, p = 0.0081, Figure 6B), and the length of the neurites measured as total length per neuron (Student’s t-test, p = 0.0088, Figure 6C) and average length per neurite (Student’s t-test, p = 0.0040, Figure 6D) was longer. In addition, the ramification index, indicating the complexity of the neurite net, increased (Student’s t-test, p = 0.0047, Figure 6E). Representative pictures of beta-III-tubulin-stained cultures at 7 DIV isolated from CTRL animals and PFBA-exposed rats are included in Figure 5 (Figure 6F). Even though the analysis of the beta-III-tubulin positive neurites showed an increased complexity of the net, the system resulted in less development than the control when analyzing the marker specificity of the mature neurites. MAP2 is a microtubule-stabilizing protein associated with the late neural maturation stage (Lesuisse and Martin, 2002).

In contrast, MAP2-positive neurites were significantly less numerous (Student’s t-test, p = 0.0154, Figure 6G) and shorter (neurite total length, Student’s t-test, p = 0.0111, Figure 6H; neurite average length, Student’s t-test, p = 0.0142; Figure 6I) in the treated groups than in the control culture. In addition, the analysis of the ramification index of the neurites showed a decreased complexity of the net (Student’s t-test, p = 0.0257, Figure 6J). Representative pictures of MAP2-stained cultures isolated from CTRL animals and rats exposed to PFBA are included in Figure 6 (Figure 6K).

At 21 DIV, when neuronal cultures are considered fully mature, expressing synaptic markers (Queenan et al., 2018), we studied a postsynaptic marker, PSD95, and a pre-synaptic marker, VGLUT. We used confocal microscopy to sample the marker-positive cells, and the z-stacks were reconstructed using the isosurfaces and analyzed using IMARIS software using the “spot” algorithm. PSD95 puncta per cell were significantly lower in cultures isolated from PFBA-exposed animals (Student’s t-test, p = 0.0115, Figures 6M,N), and the same reduction was quantified using the VGLUT pre-synaptic marker (Student’s t-test, p = 0.0123, Figures 6O,P).

### 3.5 GenX and PFBA H-C exposure during gestation and lactation induces a long-lasting inflammation-related gene expression pattern

The PARN-125Z qPCR array was used to investigate different genes included in the following pathways and molecular functions: myelination, T-cell activation and signaling, adaptive immunity, cytokines and chemokines, inflammation, apoptosis, cell adhesion, cellular stress, receptors, transcriptional factors, and other functions related to multiple sclerosis. The full list of genes is included in Supplementary Material (Supplementary Table S5).

**FIGURE 7:**
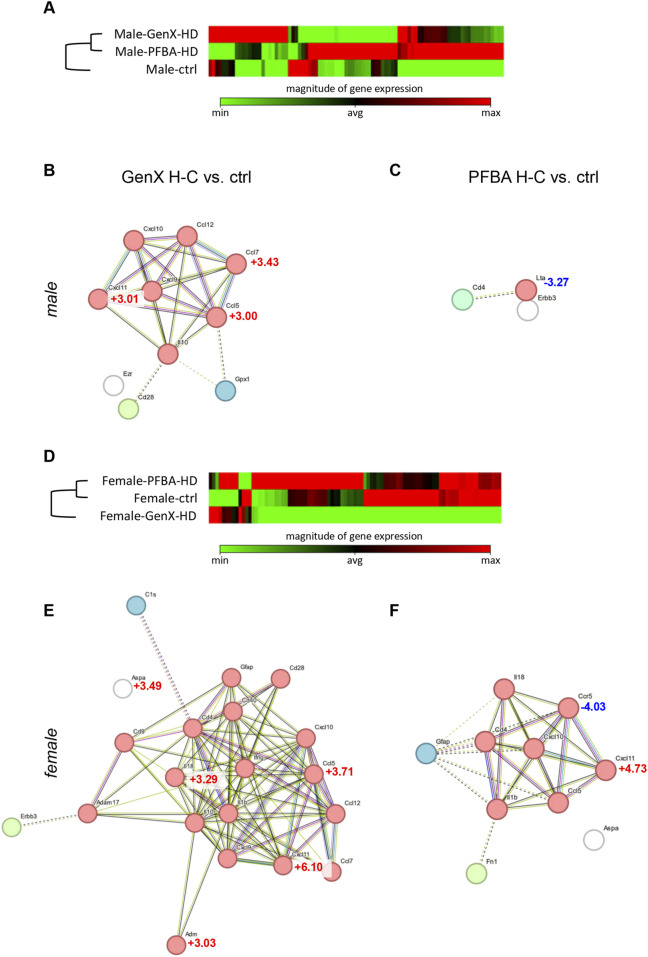
Gene expression analysis in the hippocampus of male and female animals exposed to GenX and PFBA H-C compared to control animals, using the qPCR pathway array PARN-125Z. **(A)** Clustergram representing the gene expression of the whole RT2 qPCR array in male animals. The color code is generated within the same gene between groups, as an absolute Cq value. **(B**,**C)** Functional protein association network generated by the STRING software of the identified differentially expressed genes (DEG) in male animals exposed to GenX H-C **(B)** or PFBA H-C **(C)**, compared to control animals. The network was produced using a fold of change (FoC) > 2 as a threshold, while for the DEGs showing a FoC >3, the FoC value is reported in the figure. The colors of the nodes represent the clusterization using the k-means algorithm (value = 3), and the dotted lines connect different clusters. Disconnected nodes, which have no interactions with other nodes and are not included in a cluster, are indicated in white. **(D)** Clustergram representing the gene expression of the whole RT2 qPCR array in female animals. The color code is generated within the same gene between groups, as an absolute Cq value. **(E**,**F)** Functional protein association network generated by the STRING software of the identified differentially expressed genes (DEG) in female animals exposed to GenX H-C **(E)** or PFBA H-C **(F)**, compared to control animals. The network was produced using a fold of change (FoC) > 2 as a threshold, while for the DEGs showing a FoC >3, the FoC value is reported in the figure. The colors of the nodes represent the clusterization using the k-means algorithm (value = 3), and the dotted lines connect different clusters. The disconnected nodes, which have no interactions with other nodes and are not included in a cluster, are indicated in white.

We first analyzed the differences between female and male control groups, finding only three genes slightly less expressed in females (fold of change (FoC) ≥2): CCR1 (−2.32), TGFB1 (−2.22), and VEGFA (−2.06) (Supplementary Figure S2). In male animals, both GenX H-C and PFBA H-C exposure induce important gene expression modifications in the hippocampus, leading to a separate cluster compared to the control group (Figure 7A). GenX H-C generates an increased expression in 10 genes (FoC ≥2), with three of these genes showing a FoC ≥3 (CCL5, +3.00; CXCL11, +3.01; CCL7, +3.43) (Supplementary Figure S3A,B). The proteins encoded by these DEGs are highly interconnected, forming a single cluster related to cytokines/chemokines (Figure 7B). The pathway enrichment analysis revealed that DEGs are mostly related to the immune system and inflammatory response (Gene Ontology: biological process), specifically related to cytokine and chemokine activity (Gene Ontology: molecular function) (Supplementary Figure S3C,D). PFBA H-C exposure in male animals induced the significant modification in gene expression (FoC ≥2) of only three genes, two upregulated (CD4, +2.17; ERBB3, +2.03) and one downregulated (LTA, −3.27) (Supplementary Figure S3E,F) (Figure 7C). In addition, the proteins encoded by these genes are directly involved in regulating the inflammatory response.

In female animals, GenX H-C and PFBA H-C induce a higher level of gene expression modification, with the GenX H-C-exposed group forming a separate cluster from the PFBA H-C and control groups (Figure 7D). GenX H-C generates an increased expression in 20 genes (FoC ≥2), with five of these genes showing a FoC ≥3 (ADM, +3.03; ASPA, +3.49; CCL5, +3.71; CXCL11, +6.10; IL18, +3.29) (Supplementary Figure S4A,B). In the male animals, the proteins encoded by these 20 genes are highly interconnected, mainly clustering for their inflammation-related role (Figure 7E). The pathway enrichment analysis, in fact, revealed that the DEGs are mostly related to cytokine and chemokine activity (Gene Ontology, molecular function, and biological process) (Supplementary Figure S4C,D).

PFBA H-C generates an increased expression in nine genes and a downregulation in one gene (FoC ≥2), with two of these genes showing a FoC ≥3 (CXCL11, +4.73; CCR5, -4.03) (Supplementary Figure S4E,F). These genes also mainly encode for cytokines/chemokines (Figure 7F) and are related to inflammation as described by the pathway enrichment analysis (Gene Ontology, molecular function, and biological process) (Supplementary Figure S4G,H).

We then investigated other pathways related to synaptic plasticity, starting from the female groups, in which the analysis of the inflammatory pathways revealed more DEGs than the males. The PARN-125Z plate qPCR array includes genes involved in the following pathways: immediate-early response genes, late response in synaptic plasticity, long-term potentiation, long-term depression, cell adhesion molecules, extracellular matrix molecules, CREB cofactors, neuronal receptors, postsynaptic density, and other synaptic plasticity genes (Supplementary Table S6). No differences were found in the gene expression level of these 84 genes, with only a few genes at the limit of the significant change threshold (FoC ≥2): GRM8 for GenX exposure (FoC = 2.00) and NFKBIB for PFBA exposure (FoC = 2.09) (Supplementary Figure S5).

## 4 Discussion

Human fetuses are inevitably exposed to mixtures of perfluoroalkyl substances (PFAS) throughout gestation. These compounds have been detected in virtually all examined tissues and biological fluids, including the liver, lungs, heart, central nervous system, and adipose tissue (Rappazzo et al., 2017; Mamsen et al., 2019). Developmental exposure to long-chain PFAS has been associated with cognitive impairments and behavioral alterations in both humans (Ames et al., 2025; Liu et al., 2024) and experimental animals (Bharal et al., 2024; He et al., 2025). Although replacement compounds such as PFOA alternatives were initially considered less toxic and less bioaccumulative (Bowman, 2015; Blake and Fenton, 2020), growing evidence shows that emerging short-chain PFAS are widespread in the environment. For instance, GenX has been reported in soil, grass, leaves, surface water, wildlife, domestic animals, and in human plasma (Yang et al., 2022). Similarly, PFBA has been detected at ng/mL levels in the serum of exposed populations, both from occupational settings and the general population (EFSA Panel on Contaminants in the Food Chain, 2020). These findings raise important questions regarding the potential developmental impact of these compounds.

In this study, we provide the first experimental evidence that maternal exposure to GenX and PFBA affects the neurocognitive maturation of rat offspring. Two exposure levels were tested, which were selected based on developmental toxicity studies in rodents to reflect environmentally relevant ranges (Butenhoff et al., 2012). Dams were exposed before mating, throughout gestation, and during lactation. Behavioral testing revealed significant impairments in spatial learning and memory in offspring of both sexes, as assessed by the Morris water maze. Exposed progeny showed reduced ability to use distal cues to locate the hidden platform and impaired cognitive flexibility during reversal learning.

Because only negligible levels of these compounds accumulate in the brain, the observed behavioral alterations are more likely related to the developmental disruption of neuronal processes. To test this hypothesis, we examined the maturation of fetal hippocampal neurons, a region essential for learning and memory. *In vitro* analyses demonstrated a significant delay in neuronal differentiation, with reduced expression of structural (MAP2) and synaptic (PSD95, VGLUT) markers. *In vivo*, hippocampal neurogenesis was also disrupted: nestin was upregulated while doublecortin was downregulated, consistent with delayed maturation (Kempermann et al., 2015). Moreover, gene expression profiling revealed persistent dysregulation of several neuroinflammatory pathways in adult offspring, suggesting long-term molecular alterations underlying the behavioral impairments.

### 4.1 GenX and PFBA bioaccumulation in tissues

Chemical analysis revealed slightly lower concentrations of GenX and PFBA in pellets than expected (Table 1). Thus, instead of 0.5 mg/kg bw/day and 5 mg/kg bw/day, actual exposures were 0.2 mg/kg bw/day and 1.0 mg/kg bw/day for GenX, and 0.4 mg/kg bw/day and 4.8 mg/kg bw/day for PFBA. The control diet contained trace background levels of these chemicals (0.80 μg/kg PFBA; 0.65 μg/kg GenX).

Both compounds cross the placenta (Cai et al., 2020; Fromme et al., 2010), are detectable in cord blood (Morello-Frosch et al., 2016) and breast milk (Zheng et al., 2021; Su et al., 2025), and display half-lives from a few days to approximately 1 month in humans and rodents (Olsen et al., 2007; Russell et al., 2013; Lv et al., 2024). In our study, pups were therefore indirectly exposed during a highly vulnerable period of brain development *via* placental transfer and maternal milk.

High-dose GenX- and PFBA-exposed pups showed transiently increased body weight, returning to control levels by the end of the observation period, consistent with previous findings of PFAS-induced metabolic alterations (Sen et al., 2024; Coperchini et al., 2021; Li et al., 2024).

At sacrifice (12 weeks post-weaning), GenX residues were undetectable, consistent with its rapid elimination and poor bioaccumulation (Brunn et al., 2023; Gannon et al., 2016). Similarly, negligible accumulation (<0.011%) has been reported in mice exposed to 100 mg/kg/day (Kirkwood-Donelson et al., 2024). Yet, GenX significantly altered hepatic lipid profiles, indicating that metabolic effects may occur despite limited accumulation (Kirkwood-Donelson et al., 2024).

In contrast, PFBA residues were detected in all tissues, including the brains of the high-dose groups. In particular, we observed sex-related differences in PFBA distribution, in line with the well-documented sex-dependent toxicokinetics of PFAS in rats. In adult rats, hepatic accumulation and effects of PFBA are more pronounced in males, in association with longer serum half-lives and higher systemic exposure compared to females (Butenhoff et al., 2012; Davis et al., 2022). These differences are largely driven by sex-dependent expression of organic anion transporting polypeptides (Oatp family) and other hepatic uptake transporters regulated by sex hormones (Hou et al., 2014). Consequently, male rats exhibit a greater hepatic uptake and retention of short-chain PFAS, including PFBA, than females. Conversely, renal clearance of PFAS is generally more active in females, due to higher expression and activity of renal organic anion transporters (Oat/Oatp) and hormonal regulation (Kudo et al., 2002; Kim et al., 2016). This results in faster systemic elimination but transiently higher renal tissue burdens in females during the elimination phase. In our study, we analyzed tissues from adult rats that were exposed to PFAS *in utero* and during lactation. Thus, the observed PFBA tissue concentrations reflect the combined effect of early-life exposure and sex-dependent toxicokinetics operating during growth and maturation. It is plausible that the maternal exposure established an initial body burden, subsequently modulated by sex-related differences in distribution and clearance, providing a mechanistically coherent explanation for the pattern observed in adult offspring.

Previous studies reported PFBA mainly in peripheral organs and fluids (Chang et al., 2008; Butenhoff et al., 2012), but recent evidence shows its presence in roe deer livers (Draghi et al., 2024) and in human cerebrospinal fluid (mean 0.24 ng/mL; Shang et al., 2023). The CSF/serum ratio >1 confirms PFBA’s high ability to cross the blood–brain barrier.

We measured hormonal levels at sacrifice, observing a dramatic reduction of testosterone in high-dose PFBA- and GenX-exposed males, as well as a significant decrease of progesterone in high-dose GenX-exposed females. Notably, these effects persisted 12 weeks after withdrawal. Conversely, T3 and T4 levels were almost normal, consistent with the known thyroid-disrupting effect of PFAS and its reversibility after withdrawal (Preston et al., 2018; Rubinoff and Fireman, 1989).

### 4.2 Exposure to GenX and PFBA alters neurobehavioral performance

Rats were indirectly exposed to GenX and PFBA during gestation *via* placental blood and breast milk. They were then tested for complex behaviors in adulthood when GenX was no longer present in the brain, and PFBA was only present in the brains of the H-C-exposed rats. This experimental schedule is novel in that it: (i) includes short-chain PFAS, a category of compounds not yet investigated in mammals with regard to neurodevelopment; (ii) is based on low-concentration exposure; (iii) includes the gestational and lactation periods; and (iv) analyzes complex behaviors in adult rats after a long period of exposure to contaminants has ceased, thus mimicking human developmental exposure.

We analyzed complex behaviors (locomotion, learning, and memory) as core indicators of neurodevelopmental toxicity. In the open-field test, PFBA- and GenX low-concentration-exposed male and female rats exhibited a higher distance and speed. This effect was absent in high-concentration-exposed rats, and this discrepancy could be explained by the non-monotonic dose-response relationships (NMDRs), which describe situations where the direction of a biological effect changes across the dose range, often resulting in U- or inverted U-shaped curves, and have been widely reported for endocrine-active substances (Vandenberg et al., 2012; National Toxicology Program, 2016). Such NMDR patterns are reported for PFAS toxicology, including hormone signaling and neurodevelopment (Kjeldsen and Bonefeld-Jørgensen, 2013).

No changes in the cerebellum-dependent rotarod test were observed. Notably, cerebellar involvement has been described in rodent and zebrafish models following exposure to long-chain PFAS such as PFOS (Ninomiya et al., 2022; Zoodsma et al., 2023).

The most significant outcome of this study was the severe impairment of learning, memory, and orientation in both male and female adult rats, as determined by the Morris water maze (MWM) test, which is a widely used, hippocampal-dependent task (Vorhees and Williams, 2006). Deficits were observed in both the memory and reversal phases, which assess cognitive flexibility and the capacity to unlearn and relearn spatial information. These deficits were more pronounced in GenX H-C rats. However, the study design does not include a statistical comparison between the two contaminants.

To the best of our knowledge, there are very little experimental data available on the effects of short-chain PFAS exposure on the development and maturation of cognitive domains. Our results differ from those of Marchese et al. (2024), who observed delayed neurological reflexes and altered locomotion but no memory effects in the novel object recognition (NOR) test. Differences in exposure timing (ending at birth in Marchese’s study vs. extending through lactation in our study) may explain this discrepancy because the early postnatal period corresponding to lactation is critical for synaptogenesis and hippocampal circuit refinement (Semple et al., 2013; Winters et al., 2008; Lissner et al., 2021). Similarly, Plunk et al. (2025) reported male-specific behavioral effects after gestational and postnatal PFHxA exposure, with tissue levels returning to baseline after exposure, consistent with our findings.

Overall, the behavioral outcomes observed here align with other PFAS studies: developmental exposure to short- and long-chain PFAS has been linked to persistent impairments in locomotion, learning, and memory (Guo et al., 2021; He et al., 2025).

### 4.3 Possible mechanisms

Behavioral results indicated persistent effects of maternal PFBA and GenX exposure on offspring, observed in adulthood despite the absence (GenX) or near absence (PFBA) of residues in the brain. This suggests that both compounds interfere with brain development during critical periods, leaving permanent functional consequences. Moreover, these behavioral alterations are consistent with a hippocampal-dependent mechanism.

We then directly tested this hypothesis by analyzing *ex vivo* maturation of hippocampal neurons produced by animals exposed to PFBA. Our cultures confirmed delayed neuronal maturation and impaired synaptic development. These alterations are consistent with known PFAS effects such as oxidative stress and disrupted Ca^2+^ signaling (Tukker et al., 2020; Li et al., 2024). Importantly, delayed maturation of hippocampal neurons provides a plausible explanation for the learning and memory deficits observed in the Morris water maze, as hippocampal circuit integrity is essential for spatial navigation and cognitive flexibility (Vorhees and Williams, 2006; Laeremans et al., 2015).

*In vivo*, reduced doublecortin expression in the dentate gyrus of adult males exposed to PFBA further indicated impaired adult neurogenesis. Because doublecortin-positive neurons contribute to synaptic plasticity and spatial memory (Toda and Gage, 2018; Gil-Mohapel et al., 2013), this finding directly supports the neurodevelopmental hypothesis for PFAS-dependent cognitive impairments detected in MWM tasks (Vorhees and Williams, 2006; Anacker and Hen, 2017; Hernández-Mercado and Zepeda, 2022). The sex-specific pattern (deficits stronger in males) may be partly explained by the dramatic testosterone depletion that normally promotes survival of newborn hippocampal neurons (Duarte-Guterman et al., 2019; Spritzer and Roy, 2020). Testosterone also acts on neuromodulators that play a crucial role in learning, memory, and synaptic plasticity, via genomic and membrane receptor mechanisms (Spritzer et al., 2011). A decline in learning ability is, in fact, observed in testosterone-deprived rats, associated with dendritic neurite stability by contracting MAP2 dynamics in dendritic spines (Muthu et al., 2022a) and neurotrophin-4 expression in the hippocampus (Muthu et al., 2022b). Thus, altered hormonal profiles in dams and offspring (Gaillard et al., 2025) could exacerbate neurogenic deficits and contribute to behavioral differences.

Gene expression analysis revealed a persistent pro-inflammatory molecular signature in the hippocampus of animals exposed to PFBA or GenX in both sexes, with stronger effects in GenX high-dose groups. Neuroinflammation is known to interfere with neuronal maturation and synaptic efficiency (Middelkoop and Hol, 2011; Wasel et al., 2022) and thus may contribute to the observed long-term memory and learning impairments (Laeremans et al., 2015; Tukker et al., 2020). Additional sex-specific alterations, such as GFAP upregulation (astrocytic activation; Middelkoop and Hol, 2011), ASPA overexpression (myelin lipid synthesis; Grønbæk-Thygesen and Hartmann-Petersen, 2024), and ADM induction (stress and anxiety regulation; Li et al., 2020), further suggest mechanisms linking molecular dysregulation to the behavioral phenotypes.

Taken together, these mechanistic findings provide a coherent explanation for the behavioral results: PFBA alters neuronal maturation, neurogenesis, and inflammatory pathways in the hippocampus, leading to the observed deficits in learning, memory, and cognitive flexibility (Vorhees and Williams, 2006; Gil-Mohapel et al., 2013; Anacker and Hen, 2017). This evidence suggests a possible mechanism for short-chain PFAS, strengthening the biological plausibility of our behavioral data and highlighting the neurodevelopmental risks posed by GenX and PFBA.

### 4.4 Study limitations and conclusions

This study has some limitations that should be acknowledged. In our experimental schedule, we did not include the measurement of PFAS and/or hormone concentrations immediately after the end of dietary exposure. Such data would help to discriminate between direct neurotoxic effects of GenX and PFBA in the brain and indirect consequences mediated by maternal or fetal endocrine alterations. Moreover, a complete hormonal profile at weaning was not available, limiting the interpretation of endocrine contributions to the observed outcomes.

Moreover, we explored the neural differentiation by *ex vivo* cultures in the PFBA H-C group only. A comparative study of other short-chain PFAS will be the focus of further studies.

We encountered technical limitations in the sensorimotor integration testing (rotarod), as our apparatus is suitable for the testing of small rats (up to 250 gr), while adult male rats weighed >350 gr.

Finally, the quantification of PFBA H-C residue in tissues was possible in only two replicates, and these results must be confirmed with a larger number of animals.

It is important to underline that our study was restricted to two representative short-chain PFAS, and results cannot be directly generalized to the entire class of emerging compounds.

Despite these limitations, the integration of behavioral, cellular, and molecular endpoints across multiple critical developmental windows (pre-mating, gestation, and lactation) provides robust and convergent evidence that short-chain PFAS can interfere with hippocampal development and cognitive function. By demonstrating persistent impairments in learning, memory, and cognitive flexibility together with delayed neuronal maturation, impaired neurogenesis, and chronic neuroinflammation, our findings strengthen the biological plausibility of human epidemiological associations and highlight the neurodevelopmental risks of GenX and PFBA. These results raise urgent concerns regarding the assumption that short-chain PFAS are safe alternatives to legacy compounds and call for precautionary approaches in their regulation and use.

## Data Availability

The datasets comprising all data that were generated and analyzed for this study can be found in the AMS Acta repository (https://amsacta.unibo.it/id/eprint/8395).
