# Supplementary material for: Short-chain PFAS exposure during gestation and breastfeeding alters learning and memory in adulthood: possible mechanisms related to brain development
Source: Front Toxicol. 2026 Jan 8;7:1702330. doi: 10.3389/ftox.2025.1702330 (PMC12823842; doi:10.3389/ftox.2025.1702330)
Supplement: Supplementary file 1 [file Table1.docx]

Supplementary Material

# Supplementary Materials and methods

# Analytical method validation and quality controls

# Analytical method was fully validated and the following parameters were evaluated: specificity, linearity, recovery, repeatability, limit of quantification (LOQ). Specificity was assessed in blank matrix by verifying the absence of signal higher than 30% of the LOQ level. Linearity was studied by means of calibration curves constructed with seven levels (including zero level) within the range of 0.125-7.5 µg/L (corresponding to 0.025 – 1.5 µg/kg in the sample). Calibration curves were built by plotting the instrument signal versus the analyte concentration. Linear regression analysis was carried out, and the linear calibration model was verified by correlation coefficients (Pearson’s R) better than 0.992 and by the Mandel test. Deviation from back-calculated concentration from true concentration was less or equal to 20%. The matrix effect was studied by comparing the slope obtained in solvent calibrations with the corresponding matrix-matched calibration at the same concentrations, verifying the signal suppression or enhancement (not more than 20%). Trueness and precision were estimated by analyzing seven replicates at three concentration levels for PFBA (50 - 100 - 1000 ng/kg) and two levels (100 -1000 ng/kg) for GenX. LOQ was estimated as the lowest spike level meeting the method criteria for recovery (70–120%) and precision (CV% ≤ 20%). Two negative quality control (QC) samples, one that included all analytical steps in the absence of the original matrix (QC-), and a second consisting of a fully processed blank matrix sample (QCM-), and two positive QC samples, i.e., two blank matrix matched samples, spiked at 0.05 μg/kg for PFBA and 0.10 μg/kg for GenX were processed and analyzed in each analytical series to verify absence of contamination and method performance. Whenever the concentration of the analysed sample was out the linearity range of the method, samples were properly diluted with the same matrix extract used to prepare negative quality control (QCM-).

# Supplementary table S1. Instrumental mass spectrometry settings for target compounds

| Compound | Precursor ion  (m/z) | Product ion 1  (m/z) | Collision  energy (eV) | Product ion 2  (m/z) | Collision  energy (eV) | Retention time  (min) | Internal  standard |
| --- | --- | --- | --- | --- | --- | --- | --- |
| PFBA | 213.0 | 169.1 | -14 |  |  | 5.1 | MPFBA |
| MPFBA | 217.0 | 172.0 | -14 |  |  | 5.1 |  |
| Gen X | 328.5 | 284.9 | -8 | 168.9 | -17 | 10.3 | M5PFHxA |
| M5PFHxA | 318.0 | 273.0 | -13 |  |  | 10.0 |  |

# Supplementary table S2. Method recovery and repeatability, intraday measurements (blank sample spiked with four different concentrations, seven replicates for each concentration).

|  |  | Recovery (%)  (n = 7) | | | | Precision (CV %)  (n = 7) | | | |
| --- | --- | --- | --- | --- | --- | --- | --- | --- | --- |
| Compound | Nominal conc. (µg/kg) | Liver | Kidney | Brain | Thyroid | Liver | Kidney | Brain | Thyroid |
| PFBA | 0.050* | 108.9 | 85.6 | 101.5 | 99.8 | 16.1 | 11.6 | 7.6 | 8.9 |
|  | 0.10 | 97.2 | 81.9 | 100.3 | 93.9 | 10.2 | 13.7 | 8.1 | 10.1 |
|  | 1.0 | 87.5 | 82.3 | 96.3 | 88.3 | 9.8 | 9.6 | 5.3 | 7.9 |
| Gen X | 0.10* | 101.1 | 92.6 | 99.4 | 87.3 | 17.2 | 19.3 | 14.3 | 9.3 |
|  | 1.0 | 95.6 | 94.3 | 87.3 | 81.6 | 15.3 | 13.2 | 14.9 | 8.4 |

# *Limit of Quantification

# Supplementary table S3. Samples and pooled samples of group A submitted to chemical analysis.

| Group | Matrix | Family | Sample/pool (individuals) | Sex | Sample ID |
| --- | --- | --- | --- | --- | --- |
| A | Liver/  Kidney/  Brain | A1 | Mother | F | A1 |
|  |  |  | Puppies (A1.1, A1.2) | F | A1-PF |
|  |  |  | Puppies (A1.3, A1.4) | M | A1-PM |
|  |  | A2 | Mother | F | A2 |
|  |  |  | Puppies (A2.1, A2.4, A2.5, A2.6) | F | A2-PF |
|  |  |  | Puppies (A2.2, A2.3, A2.7, A2.8, A2.9, A2.10, A2.11) | M | A2-PM |
|  |  | A3 | Mother | F | A3 |
|  |  | A4 | Mother | F | A4 |
|  |  |  | Puppies (A4.3, A4.4, A4.6, A4.7) | F | A4-PF |
|  |  |  | Puppies (A4.1, A4.2, A4.5, A4.8) | M | A4-PM |
|  |  | A5 | Mother | F | A5 |
|  |  |  | Puppies (A5.1, A5.2, A5.4, A5.5, A5.6, A5.7) | F | A5-PF |
|  |  |  | Puppies (A5.3, A5.8) | M | A5-PM |
|  | Thyroid | A1, A2, A3, A4, A5 | Mothers (A1, A2, A3, A4, A5) | F | A-T |
|  |  |  | Puppies (A1.1, A1.2, A2.1, A2.4, A2.5, A2.6, A4.3, A4.4, A4.6, A4.7, A5.1, A5.2, A5.4, A5.5, A5.6, A5.7) | F | A-T-F |
|  |  |  | Puppies (A1.3, A1.4, A2.2, A2.3, A2.7, A2.8, A2.9, A2.10, A2.11, A4.1, A4.2, A4.5, A4.8, A5.3, A5.8) | M | A-T-M |

# Supplementary table S4. Samples and pooled samples of group B1submitted to chemical analysis.

| Group | Matrix | Family | Sample/pool (individuals) | Sex | Sample ID |
| --- | --- | --- | --- | --- | --- |
| B1 | Liver/  Kidney/  Brain | B1.1 | Mother | F | B1.1 |
|  |  |  | Puppies (B1.1.1, B1.1.2, B1.1.4, B1.1.6, B1.1.7, B1.1.11, B1.1.13 | F | B1.1-PF |
|  |  |  | Puppies (B1.1.3, B1.1.5, B1.1.8, B1.1.9, B1.1.10, B1.1.12) | M | B1.1-PM |
|  |  | B1.2 | Mother | F | B1.2 |
|  |  |  | Puppies (B1.2.1, B1.2.5, B1.2.6, B1.2.7, B1.2.8, B1.2.9) | F | B1.2-PF |
|  |  |  | Puppies (B1.2.2, B1.2.3, B1.2.4, B1.2.10, B1.2.11) | M | B1.2-PM |
|  |  | B1.3 | Mother | F | B1.3 |
|  |  |  | Puppies (B1.3.2, B1.3.4) | F | B1.3-PF |
|  |  |  | Puppies (B1.3.1, B1.3.3, B1.3.5, B1.3.6) | M | B1.3-PM |
|  | Thyroid | B1.1, B1.2, B1.3 | Mother (B1.1, B1.2, B1.3) | F | B1-T |
|  |  |  | Puppies (B1.1.1, B1.1.2, B1.1.4, B1.1.6, B1.1.7, B1.1.11, B1.1.13, B1.2.1, B1.2.5, B1.2.6, B1.2.7, B1.2.8, B1.2.9, B1.3.2, B1.3.4) | F | B1-T-F |
|  |  |  | Puppies (B1.1.3, B1.1.5, B1.1.8, B1.1.9, B1.1.10, B1.1.12, B1.2.2, B1.2.3, B1.2.4, B1.2.10, B1.2.11, B1.3.1, B1.3.3, B1.3.5, B1.3.6) | M | B1-T-M |

# Supplementary table S5. Samples and pooled samples of group C1 submitted to chemical analysis.

| Group | Matrix | Family | Sample/pool (individuals) | Sex | Sample ID |
| --- | --- | --- | --- | --- | --- |
| C1 | Liver/  Kidney/  Brain | C1.1 | Mother | F | C1.1 |
|  |  |  | Puppies (C1.1.1 + C1.1.3 +C1.1.6) | F | C1.1-PF |
|  |  |  | Puppies (C1.1.2 +C1.1.5) | M | C1.1-PM |
|  |  | C1.2 | Mother | F | C1.2 |
|  |  |  | Puppies (C1.2.2, C1.2.3, C1.2.6, C1.2.7, C1.2.10) | F | C1.2-PF |
|  |  |  | Puppies (C1.2.1, C1.2.4, C1.2.8, C1.2.7, C1.2.9) | M | C1.2-PM |
|  |  | C1.3 | Mother | F | C1.3 |
|  |  |  | Puppy (C1.3.1) | F | C1.3-PF |
|  |  |  | Puppies (C1.3.2, C1.3.3, C1.3.4, C1.3.5, C1.3.6, C1.3.8) | M | C1.3-PM |
|  |  | C1.4 | Mother | F | C1.4 |
|  |  |  | Puppies (C1.4.1, C1.4.2, C1.4.3) | M | C1.4-PM |
|  | Thyroid | C1.1, C1.2, C1.3, C1.4 | Mother (C.1.1, C1.2, C1.3, C1.4) | F | C1-T |
|  |  |  | Puppies (C1.1.1, C1.1.3, C1.1.6, C1.2.2, C1.2.3, C1.2.6, C1.2.7, C1.2.10) | F | C1-PF-T |
|  |  |  | Puppies (C1.1.2 +C1.1.5 C1.2.1, C1.2.4, C1.2.8, C1.2.7, C1.2.9, C1.3.2, C1.3.3, C1.3.4, C1.3.5, C1.3.6, C1.3.8) | M | C1-PM-T |

# Supplementary table S6. Samples and pooled samples of group B2 submitted to chemical analysis.

| Group | Matrix | Family | Sample/pool (individuals) | Sex | Sample ID |
| --- | --- | --- | --- | --- | --- |
| B2 | Liver/  Kidney/  Brain* | B2.2 | Mother | F | B2.2 |
|  |  |  | Puppies (B2.2.1, B2.2.4, B2.2.6, B2.2.10, B2.2.12, B2.2.14, B2.2.17, B2.2.20) | F | B2.2-PF |
|  |  |  | Puppies (B2.2.7, B2.2.8, B2.2.6, B2.2.15, B2.2.19) | M | B2.2-PM |
|  |  | B2.3 | Mother | F | B2.3 |
|  |  |  | Puppies (B2.3.1, B2.3.5, B2.3.6, B2.3.8, B2.3.9, B2.3.10) | F | B2.3-PF |
|  |  |  | Puppies (B2.3.2, B2.3.3, B2.3.4, B2.3.7, B2.3.11, B2.3.12) | M | B2.3-PM |
|  |  | B2.4 | Mother | F | B2.4 |
|  |  |  | Puppies (B2.4.5, B2.4.10) | F | B2.4-PF |
|  |  |  | Puppies (B2.4.1, B2.4.3, B2.4.6, B2.4.8, B2.4.11, B2.4.13) | M | B2.4-PM |
|  | Thyroid | B2.2, B2.3, B2.4 | Mothers (B2.2, B2.3, B2.4) | F | B2-T |
|  |  |  | Puppies (B2.2.1, B2.2.4, B2.2.6, B2.2.10, B2.2.12, B2.2.14, B2.2.17, B2.2.20, B2.3.1, B2.3.5, B2.3.6, B2.3.8, B2.3.9, B2.3.10, B2.4.5, B2.4.10) | F | B2-PF-T |
|  |  |  | Puppies (B2.2.7, B2.2.8, B2.2.6, B2.2.15, B2.2.19, B2.3.2, B2.3.3, B2.3.4, B2.3.7, B2.3.11, B2.3.12, B2.4.1, B2.4.3, B2.4.6, B2.4.8, B2.4.11, B2.4.13) | M | B2-PM-T |

# * Brain samples of the following animals were not available for chemical analysis: B2.2.4, B2.2.1, B2.2.7, B2.3.5, B2.3.12, B2.4.5, B2.4.1, B2.4.3

# Supplementary table S7. Samples and pooled samples of group C2 submitted to chemical analysis.

| Group | Matrix | Family | Sample/pool (individuals) | Sex | Sample ID |
| --- | --- | --- | --- | --- | --- |
| C2 | Liver/  Kidney/  Brain** | C2.1 | Mother | F | C2.1 |
|  |  |  | Puppies (C2.1.1, C2.1.2, C2.1.4, C2.1.10, C2.1.11, C2.1.12, C2.1.13) | F | C2.1-PF |
|  |  |  | Puppies (C2.1.3, C2.1.5, C2.1.6, C2.1.7, C2.1.8, C2.1.9) | M | C2.1-PM |
|  |  | C2.2 | Mother | F | C2.2 |
|  |  |  | Puppies (C2.2.1, C2.2.2, C2.2.5, C2.2.6, C2.2.7, C2.2.10) | F | C2.2-PF |
|  |  |  | Puppies (C2.2.3, C2.2.4, C.2.8, C2.2.9) | M | C2.2-PM |
|  | Thyroid |  | Mothers (C2.1, C2.2) | F | C2-T |
|  |  |  | Puppies (C2.1.1, C2.1.2, C2.1.4, C2.1.10, C2.1.11, C2.1.12, C2.1.13, C2.2.1, C2.2.2, C2.2.5, C2.2.6, C2.2.7, C2.2.10) | F | C2-PF-T |
|  |  |  | Puppies (C2.1.3, C2.1.5, C2.1.6, C2.1.7, C2.1.8, C2.1.9, C2.2.3, C2.2.4, C.2.8, C2.2.9) | M | C2-PM-T |

# ** Brain samples of the following animals were not available for chemical analysis: C2.1.2, C2.1.11, C2.1.3, C2.1.6, C2.1.9, C2.2.1, C2.2.6, C2.2.8

# Supplementary table S8. Sensitivity and linearity range for hormonal assays.

| **Parameter** | **Analytical method** | **Analytical sensitivity** | **Linearity range** |
| --- | --- | --- | --- |
|  |  |  |  |
| **T3** | ECLIA | 20 ng/dL | 20-651 ng/dL |
| **T4** | ECLIA | 0.42 µg/dL | 0.42-24.86 µg/dL |
| **Progesterone** | ECLIA | 0.05 ng/mL | 0.05-60 ng/mL​ |
| **Testosterone** | ECLIA | 2.5 ng/dL | 2.5-1500 ng/dL |
